# RhCu 3D Nanoframe as a Highly Active Electrocatalyst for Oxygen Evolution Reaction under Alkaline Condition

**DOI:** 10.1002/advs.201500252

**Published:** 2015-09-25

**Authors:** Jongsik Park, Jongchan Kim, Yoojin Yang, Donghwan Yoon, Hionsuck Baik, Seungjoo Haam, Haesik Yang, Kwangyeol Lee

**Affiliations:** ^1^ Center for Molecular Spectroscopy and DynamicsInstitute for Basic Science (IBS)Department of ChemistryKorea UniversitySeoul136‐701South Korea; ^2^ Department of Chemistry and Chemistry, Institute of Functional MaterialsPusan UniversityBusan609‐735South Korea; ^3^ Korea Basic Science Institute (KBSI)Seoul136‐713South Korea; ^4^ Department of Chemical & Biomolecular EngineeringYounsei UniversitySeoul120‐749South Korea

**Keywords:** alkaline condition, core–shell, nanoframe, rhodium, water splitting catalyst

## Abstract

**One pot synthesis** of RhCu alloy truncated octahedral nanoframes, Cu@Rh core–shell nanoparticles, and a bundle of five RhCu nanowires is demonstrated. The RhCu alloy 3D nanoframe, in particular, exhibits excellent catalytic activity toward the oxygen evolution reaction under alkaline conditions.

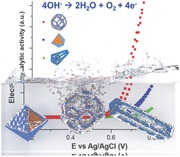

Nanoparticle structure engineering has been extensively studied in recent years for the development of active nanocatalysts in energy conversion technologies such as fuel cells.^1^, ^2^, ^3^, ^4^, ^5^, ^6^, ^7^, ^8^, ^9^ A great improvement in this area has been feasible with facet‐controlled nanoparticles and core–shell nanoparticles with lattice mismatch, which enables surface‐energy modulations.^10^, ^11^, ^12^, ^13^, ^14^, ^15^, ^16^, ^17^ Most notably, phenomenal enhancement in catalytic performance has been accomplished by framework nanostructures with dramatically increased surface area per volume, and a great deal of current interest resides on the design and synthesis of novel nanoframework structures.^18^, ^19^, ^20^, ^21^, ^22^, ^23^ Pt‐based alloy nanoframeworks constitute a majority of nanoframeworks and the application of these nanostructures is mostly confined to the fuel cell electrode oxygen reduction reaction (ORR).^24^, ^25^, ^26^, ^27^ Relatively little has been studied for the preparation of non‐Pt nanoframeworks and for the application of nanoframework structures in other important catalysis reactions such as oxygen evolution reaction (OER). Nanoparticles of metals or metal oxides of Ru, Rh, Ir, and Pt have been examined as electrocatalysts for OER, and the literature results seem to identify surface area of nanocatalysts as the most important parameter for catalytic activity.^28^, ^29^, ^30^, ^31^, ^32^ Therefore, it is intriguing to study catalytic activities of nanoframework structures for these metals, in particular non‐Pt metals, toward OER, and we have attempted to prepare nanoframework structures for Ru and Ir by using Cu as the in situ formed removable template.^33^, ^34^ However, very well‐defined nanoframeworks found for Pt‐based alloys could not be reproduced with these metals. Decomposition of Ru or Ir precursors required very high reaction temperature which would exert adverse effects on differentiating edges and facet centers. On the other hand, Rh metal precursor, namely Rh(acac)_3_, can be decomposed at much lower temperature than Ru or Ir precursors and this might lead to the formation of desired alloy nanoframework structures. Herein, we report the synthesis and application of novel RhCu nanoframe structures as effective OER catalysts under alkaline condition as well as their structure dependent‐catalytic activities.

In a typical synthesis of a RhCu truncated octahedral nanoframe (RCTOF), a slurry of rhodium acetylacetonate (Rh(acac)_3_), copper(II) acetylacetonate (Cu(acac)_2_), and stearic acid (SA) in oleylamine (OAm) was prepared in a 100 mL Schlenk tube with magnetic stirring. After being placed under vacuum at 25 °C for 10 min, the solution was charged with 1 atm CO. Then the Schlenk tube was directly placed in a hot oil bath, which was preheated to 250 °C. After being heated at the same temperature for 30 min, the reaction mixture was cooled down to room temperature with magnetic stirring. The reaction mixture, after being added 15 mL toluene and 20 mL ethanol, was centrifuged at 3500 rpm for 5 min. The resulting precipitates were further purified twice by washing with 20 mL ethanol and 15 mL toluene.

Transmission electron microscopy (TEM) and high‐resolution transmission electron microscopy (HRTEM) images of RCTOFs are shown in **Figure** **1**. The average size of RCTOF was determined as 25.2 ± 1.2 nm (see Figure S1 in the Supporting Information for size analysis). The HRTEM image in Figure 1b clearly shows the structural feature of truncated octahedral nanoframe. An ideal model of RCTOF is depicted in Figure 1c, and the purple and green planes in the model are perpendicular to the direction of 〈100〉 and 〈111〉, respectively. An enlarged HRTEM image of the nanoframe edge reveals that RCTOF is composed of both RhCu alloy phase and Rh phase. The lattice spacing of {220} plane of edge part of RCTOF is measured as 0.132 nm, which is shorter than {220}Rh (*d* = 0.135 nm) but longer than {220}Cu (*d* = 0.128 nm). However, the HRTEM image of the nanoframe vertex shows the presence of Rh phase; pure {111}Rh lattice (*d* = 0.222 nm) could be identified. X‐ray powder diffraction (XRD) pattern of RCTOF also indicates the presence of both RhCu alloy phase and Rh phase (Figure S2, Supporting Information). In order to understand the structural feature of coexisting RhCu alloy and Rh phases in detail, we analyzed the line profile data and ED pattern of RCTOF as shown in Figure S3 (Supporting Information). The line profile data of RCTOF show an interesting Rh composition fluctuation; the Rh count increases and then decreases steeply as the Rh count is collected from the outer shell to the inner shell, while the Cu count only increases at the same time. This observation is consistent with a structural model which exhibits a mainly Rh phase at the outermost part of the hollow cage and CuRh phase underneath the Rh layer. As the reaction proceeds, the Rh precursor which remains in solution would be decomposed and preferably deposited at the vertex sites of RCTOF, because vertex sites are highly active and need to be stabilized. Similar preferred growth on the vertex sites has been previously reported with other nanostructures.^35^, ^36^, ^37^ In order to better understand the structural feature of RCTOF, frame structures with different zone axes were obtained as shown in Figure 1d. The elemental mapping of RCTOF in Figure 1e shows that both Rh (green) and Cu (red) are evenly distributed over the entire nanostructure. The nanoframe structure consists of 54.2% Rh and 45.8% Cu as determined by energy dispersive X‐ray study (Figure S4, Supporting Information). Therefore, the nanoframe composition can be described as mainly composed of RhCu, spiked by minor Rh phase.

**Figure 1:**
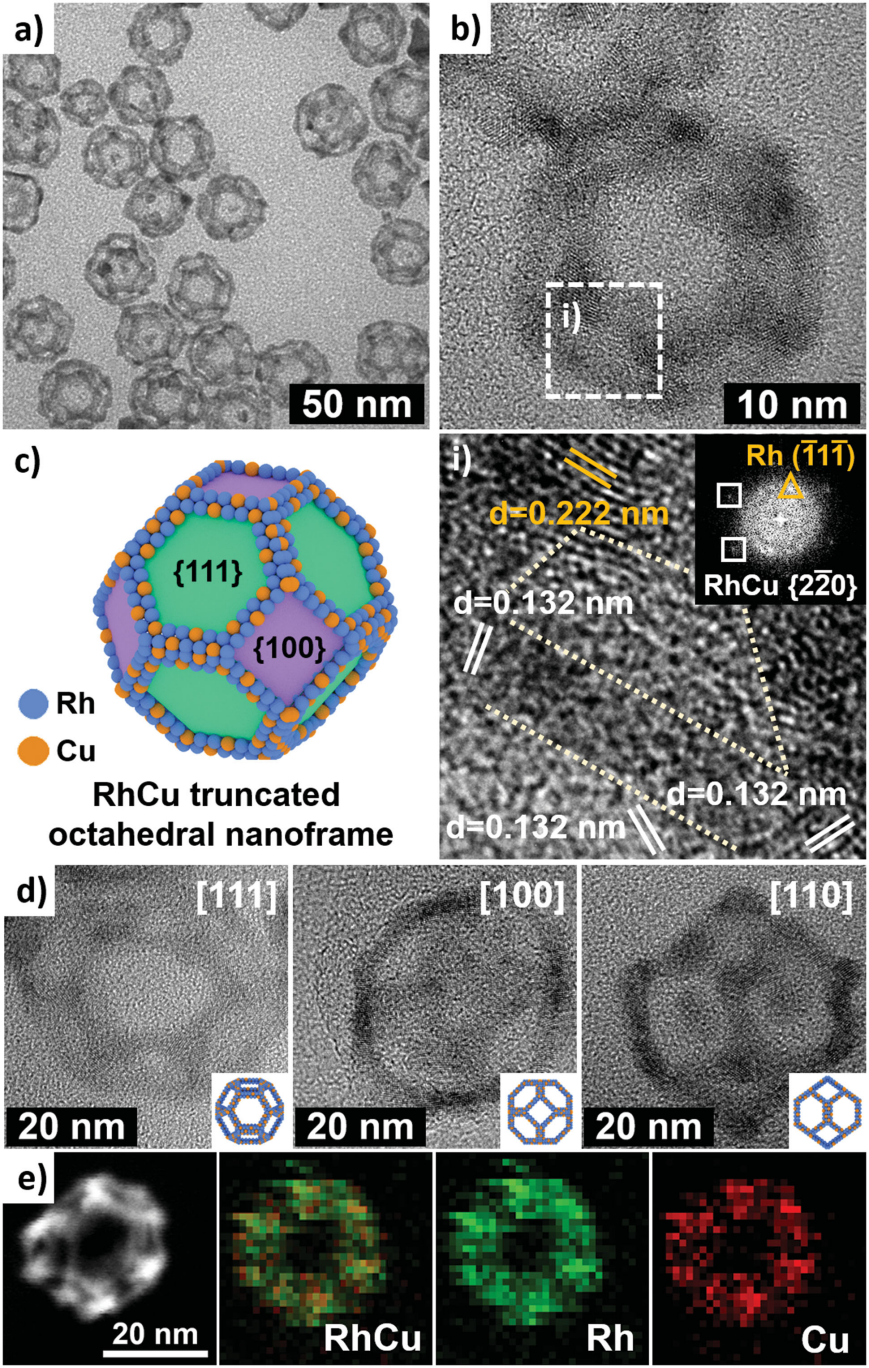
Characterization of RCTOFs. a) TEM image of RCTOF. b) HRTEM image of RCTOF. i) Enlarged HRTEM image of white marks in panel (b) (Inset: the fast Fourier transform (FFT) pattern along the zone axis of [111]). The orange and white marks in the magnified HRTEM image represent {111}Rh and {220}RhCu spacings, respectively. c) An ideal model of a RCTOF. d) HRTEM images with different orientations. The zone axes of HRTEM images are [111], [100], and [110], respectively. e) HAADF‐STEM image and corresponding elemental mapping analysis of Rh (green) and Cu (red) of the RCTOF. It is clearly shown that Rh and Cu contents are thoroughly mixed over the entire nanoframe.

To elucidate the formation mechanism of RCTOF, nanocrystals produced with different reaction durations were studied by TEM images. **Figure** **2** shows that the synthesis of RCTOF could be finished within 30 min. Nanoparticles collected after reaction for 3 min are mostly irregular‐shaped Cu nanoparticles (Figure S5, Supporting Information). Further heating of the reaction mixture converts these nanoparticles into truncated concave octahedron shapes, plausibly due to the surface etching of truncated octahedron. The hollowing out of truncated octahedral nanostructures is nearly completed at the reaction time of 13 min. Finally, well‐defined nanoframe structures of RCTOF are observed at 30 min. Because the decomposition of Rh starts after the formation of Cu nanoparticles, Galvanic replacement occurring on preexisted Cu nanoparticles seems to be an important reaction mechanism leading to the formation of hollow nanostructures.

**Figure 2:**
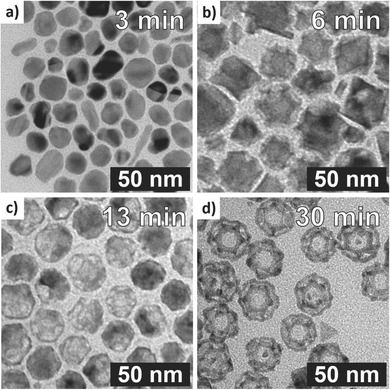
Temporal TEM images of RCTOF obtained at a) 3 min, b) 6 min, c) 13 min, and d) 30 min, respectively.

An interesting morphological development was observed when 1,2‐hexadecanediol was added to the initial reaction mixture (as shown in **Figure** **3**). Decomposition and reduction rates of Rh(acac)_3_ and Cu(acac)_2_ can be strongly facilitated by the existence of reducing agent 1,2‐hexadecanediol. Because Cu nanoparticles are formed at the initial stage and more Rh doping to the initially formed Cu seeds is feasible due to the facilitated reduction by 1,2‐hexadecanediol, the observed morphology of truncated cube appears to result from Rh doping effect on the Cu nanoparticle shape control. Impurity doping has been employed in certain cases to control nanoparticle shapes.^38^, ^39^ At the reaction time of 10 min, a truncated cube morphology is observed and hollowing out is minimal. After 30 min of reaction time, corners of RhCu nanocube become further truncated, likely due to enhanced Cu etching at vertex sites. Elemental mapping analysis in Figure 3c shows that RhCu hollow nanocubes consist of the RhCu alloy phase. High angle annular dark field ‐ scanning transmission electron microscopy (HAADF‐STEM) and line profile data (Figure 3d) indicate that the edges and facets are composed of both Rh and Cu. Constant Rh/Cu ratio was found over the entire nanoframe structure.

**Figure 3:**
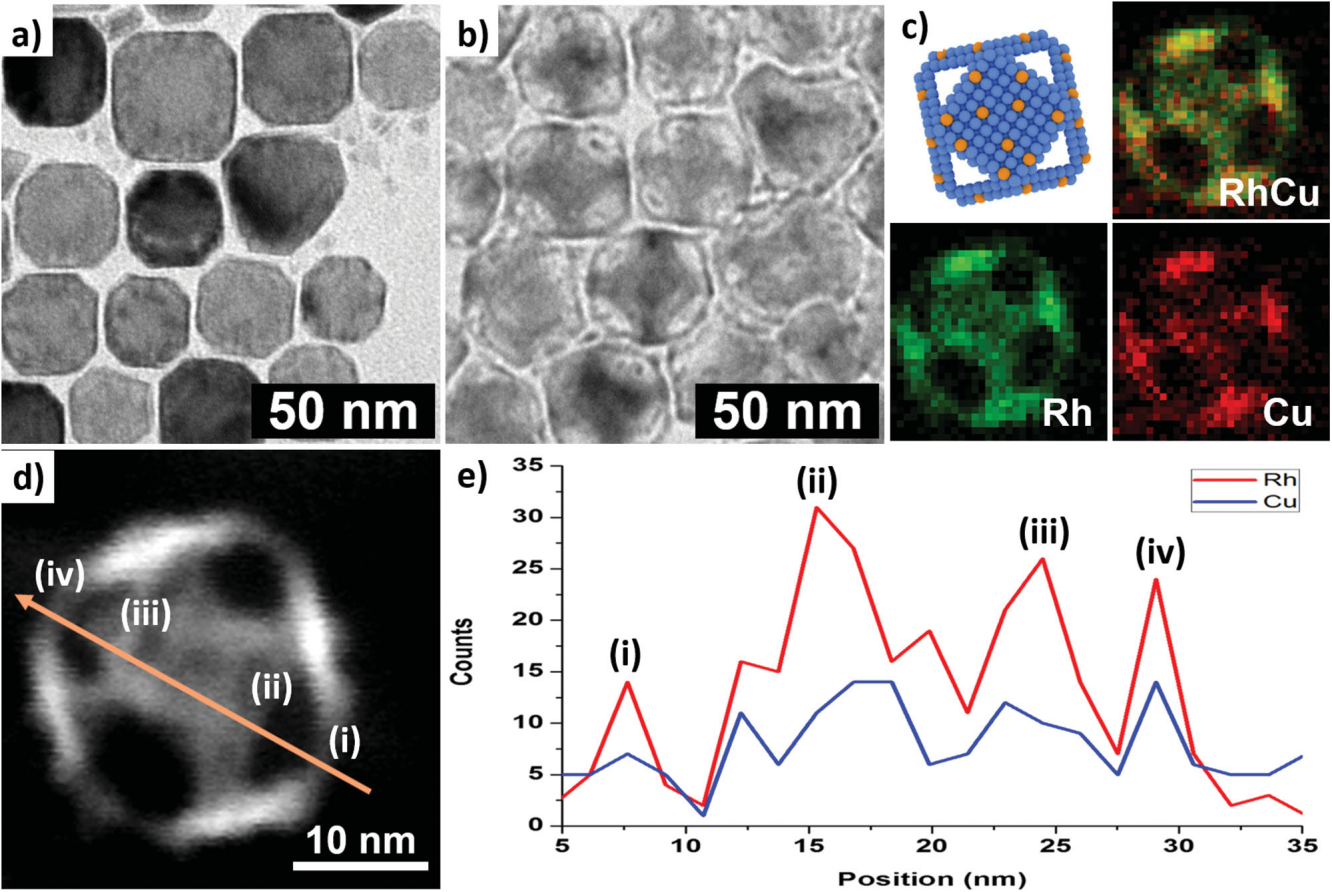
Characterization of RhCu hollow nanocube structures. TEM images of RhCu nanocube at reaction times of a) 10 min and b) 30 min, respectively. c) Ideal model of RhCu hollow nanocube and elemental mapping analysis of Rh (green) and Cu (red). d) HAADF‐STEM image of RhCu hollow nanocube. e) Line profile data for Rh (red line) and Cu (blue line) contents.

An interesting morphology of a bundle of five nanowires with both ends tied (RhCu fivefold twinned nanoframes, RCFTF) was obtained by aging the reaction mixture at 100 °C for 4 h under Ar gas prior to the above mentioned experimental procedures. The white arrows in **Figure** **4**a point to five nanowires of RCFTF. We have recently shown that aging of Pt(acac)_2_ in oleylamine (OAm) prior to thermal decomposition leads to the formation of fivefold twinned Pt nanorods,^40^ and attributed the morphology formation to the role of in situ formed OAm‐ligated Pt precursors. It is known that OAm could also coordinate with Cu ion, therefore, a Cu^+^‐OAm complex might be decomposed to form fivefold twinned Cu nanorods.^41^, ^42^ With this fivefold twinned Cu nanorod as a template, the observed nanostructure of RCFTF with both end tied might be explained. The nanostructure in Figure 4a can be ideally defined as a RhCu pentagonal prism nanoframe. To understand the detailed mechanism for the formation of nanostructure of RCFTF with both ends tied, the structural evolution was studied by examining temporal TEM images of reaction mixture as shown in Figure 4b–e. The initial formation of fivefold twinned Cu nanorods, localization of Rh phase on the edges, and selective removal of Cu by etching are clearly demonstrated. In summary, we could fine‐control RhCu nanoframe structures by controlling the initial seed shapes, mostly Cu based. There has been one previous report for Rh‐based octahedral nanoframe via formation of RhCu alloy nanoparticle and subsequent etching of Cu phase under acidic condition.^43^ One step formation of Rh‐based nanoframeworks of controlled shapes, however, is unprecedented to the best of our knowledge.

**Figure 4:**
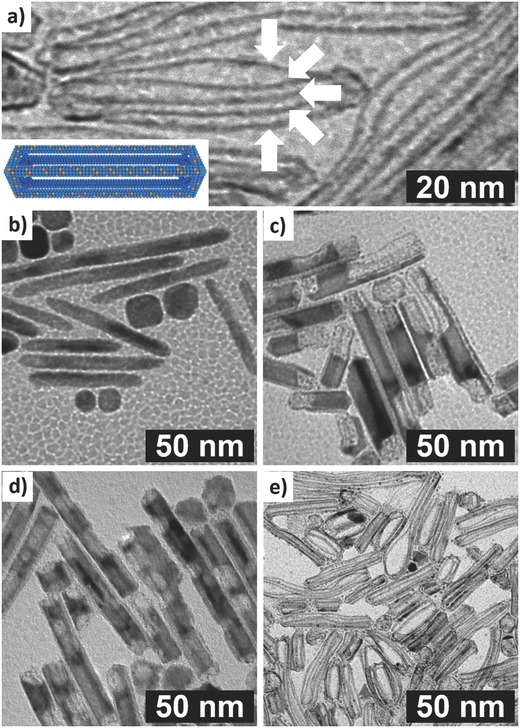
a) Magnified TEM image of RCFTF; arrows indicate the fivefold twinned nature of initial Cu‐based nanotemplates. The inset is a simplified model of RCFTF. TEM images of RCFTF at different reaction times of b) 5 min, c) 20 min, d) 40 min, and e) 1 h, respectively.

It was found that the formation of 3D nanoframe structure requires an optimal Rh to Cu ratio of 1:1. Figure S6 (Supporting Information) shows the morphological evolution of the particles prepared with controlling concentrations of Rh and Cu (Rh:Cu mole ratio was varied from 4:1 to 1:4). When the amount of used Rh precursor was higher than that of Cu precursor, Cu@Rh core–shell nanooctahedron structures (CSNO) were obtained. The high concentration of Rh precursors induced a complete encapsulation of Cu nanoparticle by Rh phase with eight {111}Rh facets, forming an octahedron. Cu atoms of Cu nanoparticles, synthesized at initial step, could not be etched out, because the growth of Rh phase might be much faster than galvanic replacement of Cu by Rh or Cu leaching process. Ultrasmall Rh nanoparticles and triangular Rh nanoplates were also synthesized because of fast Rh precursor decomposition. On the other hand, when more Cu precursors were used, higher proportion of hollow nanocages was observed. It could be explained that most of Cu precursors are consumed at a nucleation stage to form Cu‐based polyhedral nanoparticles. Subsequently, galvanic replacement of Cu by Rh occurs because of the reduction potential difference between Rh and Cu. In addition, Rh precursors are thermally decomposed on the RhCu alloy phase to form pure Rh phase. **Figure** **5** shows TEM and HRTEM images of Cu@Rh octahedron with the average size of 18.2 ± 1.6 nm prepared from Rh:Cu ratio of 4:1 (see Figure S1 in the Supporting Information for size analysis). The HRTEM image and enlarged HRTEM images in Figure 5b,c indicate that the lattice distances at octahedral vertex and facet are different. In section (i), the lattice distances suggest the presence of RhCu alloy phase; the measured lattice distances are 0.131, 0.189, and 0.210 nm and they could be indexed to the {220}, {002}, and {111} reflections of face‐centered cubic RhCu alloy, respectively. These lattice distances are shorter than those of Rh but longer than those of Cu. We could only observe Cu lattice parameters in section (ii) (yellow marks), and therefore the core is mostly composed of Cu phase. The elemental mapping data of CSNO in Figure 5c is in agreement with the nanostructure with a Cu‐rich core and Rh‐rich shell. XRD pattern of CSNO indicates the presence of both RhCu alloy phase and Cu phase (Figure S2, Supporting Information). To further understand the morphology and composition of CSNO, we obtained additional STEM images recorded at different tilting angles and energy dispersive X‐ray spectroscopy data as shown in Figure S7 (Supporting Information). The atomic composition of CSNO with octahedron morphology was found to be 21.9% Rh and 78.1% Cu. The Rh‐rich shell is very robust as judged from the fact that the morphology of CSNO is not changed even when placed in acetic acid (Figure S8, Supporting Information).

**Figure 5:**
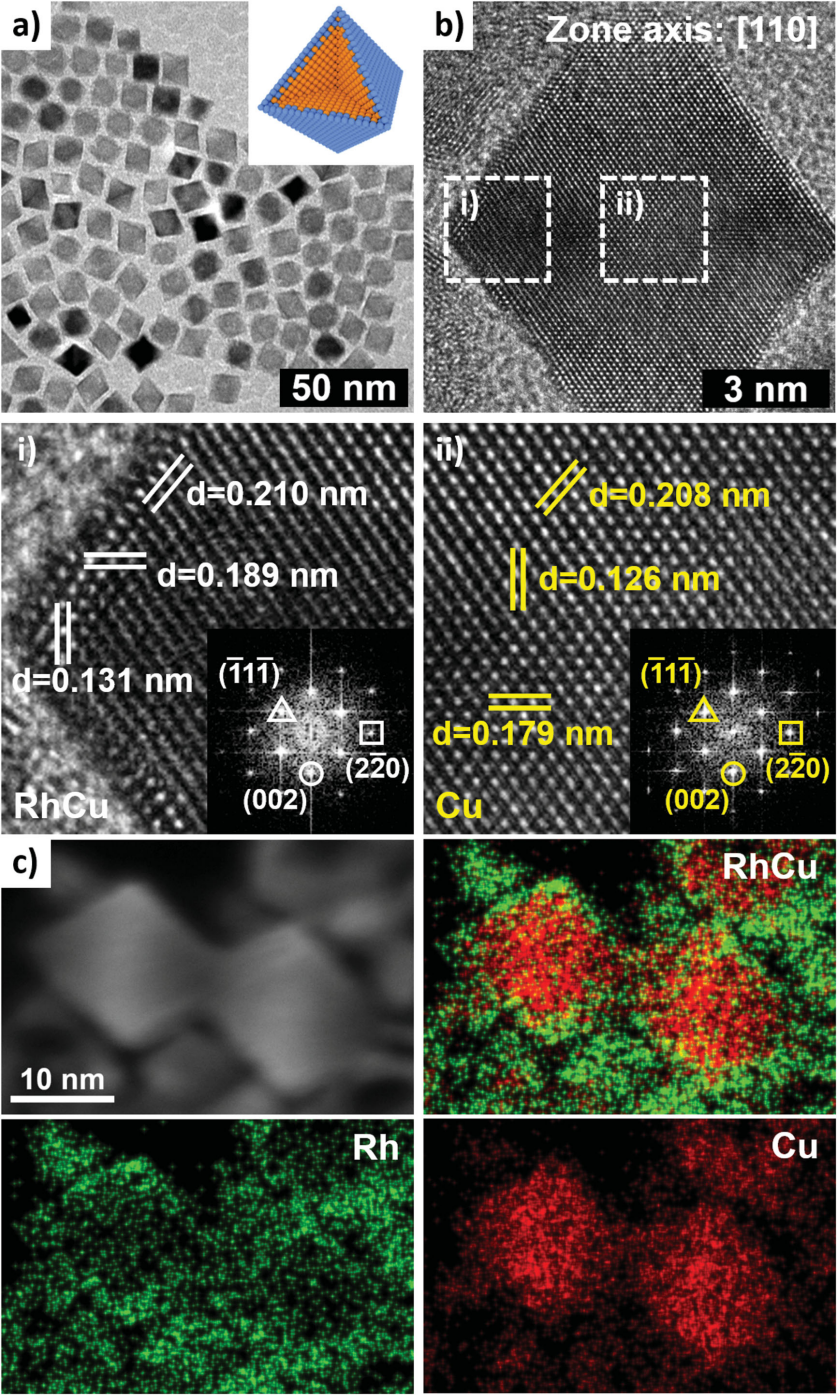
Characterization of Cu@Rh CSNO. a) TEM image of CSNO (Inset: the ideal model of CSNO). b) HRTEM image of CSNO. i,ii) Enlarged HRTEM images of white marks in panel (b). The white and yellow marks in the magnified HRTEM image represent RhCu phase and Cu phase, respectively. c) HAADF‐STEM image and corresponding elemental mapping analysis of Rh (green) and Cu (red) contents of Cu@Rh nanooctahedron. It is clearly shown that Rh component is placed on the edges of Cu‐based octahedron.

The morphology control of RhCu nanostructures is critically dependent on the amount of SA as shown in Figure S9 (Supporting Information). With SA content of 0 equiv, only small nanoparticles with poorly defined morphologies could be seen. With SA content of 1 equiv, shape‐controlled nanoframeworks are prepared. As the amount of SA is further increased, the overall size of nanoparticles is increased as well. SA has been found to slow down the decomposition of metal precursors, thereby decreasing the number of seeds, and this can lead to the overall increase in nanoparticle size due to the limited number of seeds. With the SA amount of 2 equiv, the hollowing out of the Cu phase is still observed. However, the shell thickness of hollow structures is much larger than those with 1 equiv SA condition. With 4 equiv of SA, no hollow structures are found, probably due to the unsuccessful role of Cu as a template, because the fast formation of Cu seeds would not be feasible under this condition. The overall experimental conditions for various RhCu bimetallic nanostructures are summarized in Figure S10 (Supporting Information).

Catalytic activity of new nanostructures of this study was studied for OER. OER catalysts have been studied both in acidic and alkaline conditions.^44^, ^45^ In an alkaline solution, it was previously reported that the proposed reaction mechanisms have the initial adsorption of the hydroxide ions on the metal oxide surfaces as a common step.^46^, ^47^, ^48^ After that, the removal of one proton and one electron to form a surface oxide species. Therefore, the bonding strength between the intermediate and metal also implies the OER activity. While noble metal nanostructures show a great electrocatalytic performance in acidic condition, the compositional change of alloy nanoparticles, accompanied by structural deformations due to leaching during the catalytic cycle, has been a consistent problem. Therefore, rather stable Ru or Ir oxide only has been used as electrocatalytic materials in acidic condition. In contrast, alloy nanoparticles, with leachable component in acidic condition, can be used in alkaline conditions. The major problem of OER in alkaline condition is that catalytic performances are not very different between noble and nonnoble metals. In this regard, nanoframework structures with a high surface area seem to provide the best design concept for OER catalyst for usage in alkaline solution.

Nanoparticle‐modified indiumatin oxide (ITO) electrodes were prepared by simple drop‐drying of an ethanol dispersion of Rh‐based nanostructures. **Figure** **6** shows cyclic voltammograms recorded with a bare ITO electrode and nanostructures‐modified ITO electrodes in 0.10 m NaOH and the voltage was swept from 0 to 0.8 V at a scan rate of 10 mV s^−1^. A large increase of oxidation current occurs at ≈0.53 V for our samples. The mass activity of RCTOF is largest among all examined nanostructures and is approximately twice of that of irregular shaped Rh nanoparticles, as shown in Figure S11 (Supporting Information). The specific activity of RCTOF is also larger than that of CSNO as shown in Figure S12 (Supporting Information). Also, the catalytic activity of RCTOF was much superior to those of recently reported Pt‐based nanostructures (Figure S13, Supporting Information).^49^ Metal atoms in synthesized nanostructures could be further oxidized during the first scan of cycle (Figure S14, Supporting Information). The catalytic activity, however, was stabilized from second scan, plausibly because the surface of RhCu alloy nanostructures was fully oxidized during the first scan. In addition, the RCTOF and CSNO exhibited a high durability throughout electrochemical operation; after 100 potential cycles, the catalytic performances of both nanostructures were preserved. TEM image in Figure S15 (Supporting Information) also shows that the structural feature of nanoframework has been preserved even after 100 cycles. On the other hand, the catalytic activity of CSNO and RCFTF was inferior to that of Rh nanoparticles. An important catalytic activity parameter is the surface area per volume, and it is clear that RCTOF exhibits more surface area than CSNO and Rh nanoparticles; both the interior and exterior of 3D nanoframeworks can be accessible for the catalysis. To evaluate the efficiency of the catalytic reaction, we analyze the Tafel plot derived from the polarization curves. The RCTOF exhibits a Tafel slope of 68.2 mV decade^−1^ in 0.1 m NaOH, which is lower than that of CSNO (81.2 mV decade^−1^) as shown in Figure S16 (Supporting Information). The obtained Tafel slope values suggest that RCTOF is a more favorable nanostructure than CSNO for OER. At a glance, RCFTF seems to exhibit a comparable surface area to that of RCTOF, thus not explaining its poor catalytic activity. The OER catalytic activity might also be influenced by the oxidation states of metal nanoparticle. Therefore, we obtained the X‐ray photoelectron spectroscopy (XPS) data of RCTOF, CSNO, and RCFTF (Figure S17, Supporting Information). The peaks located at 307.4, 308.3, 312.1, and 313.3 eV could be assigned to Rh(0) 3d_5/2_, Rh(III) 3d_5/2_, Rh(0) 3d_3/2_, and Rh(III) 3d_3/2_, respectively. From XPS analysis, it is found that all of our samples exhibit both Rh(0) and Rh(III) states. We suspected that a nanoparticle containing high proportion of already oxidized atoms would experience a smaller degree of oxidation during the first scan and, therefore, lesser degree of detachment from the electrode surface, which would result in poor catalytic performance. The XPS analysis of RCTOF showed more than half of Rh atoms are present in oxidized forms (63.2%) on the surface. The amount of Rh(III) oxidation states in CSNO and RCFTF, however, are 23.9%, and 32.2%, respectively, which are smaller than that of RCTOF. Therefore, high proportion of oxidized Rh atoms in RCTOF might be responsible for the observed high catalytic activity. Core–shell effects due to the lattice mismatch between the core and shell were also identified as activity‐enhancing parameter for ORR catalysis.^49^ However, CSNO with a Cu core and a RhCu shell does not seem to exhibit such catalysis enhancing property for OER. In order to study the relevance of surface area to the catalytic performance, the Bruner–Emmett–Teller (BET) surface areas of the samples were obtained by using a conventional BET single‐point N_2_ physisorption apparatus (Figure S18, Supporting Information). The samples were carefully dried at 120 °C under vacuum before analysis. The specific surface areas of RCTOF and CSNO were found to be 15.93 and 6.96 m^2^ g^−1^, respectively. The surface area of RCTOF is roughly twice of CSNO, and therefore surface area seems to play an important role in the observed catalytic activity. In short, the surface area enhanced 3D nanoframework structure with easily oxidized metal atoms might be useful in boosting the catalytic activity for OER.

**Figure 6:**
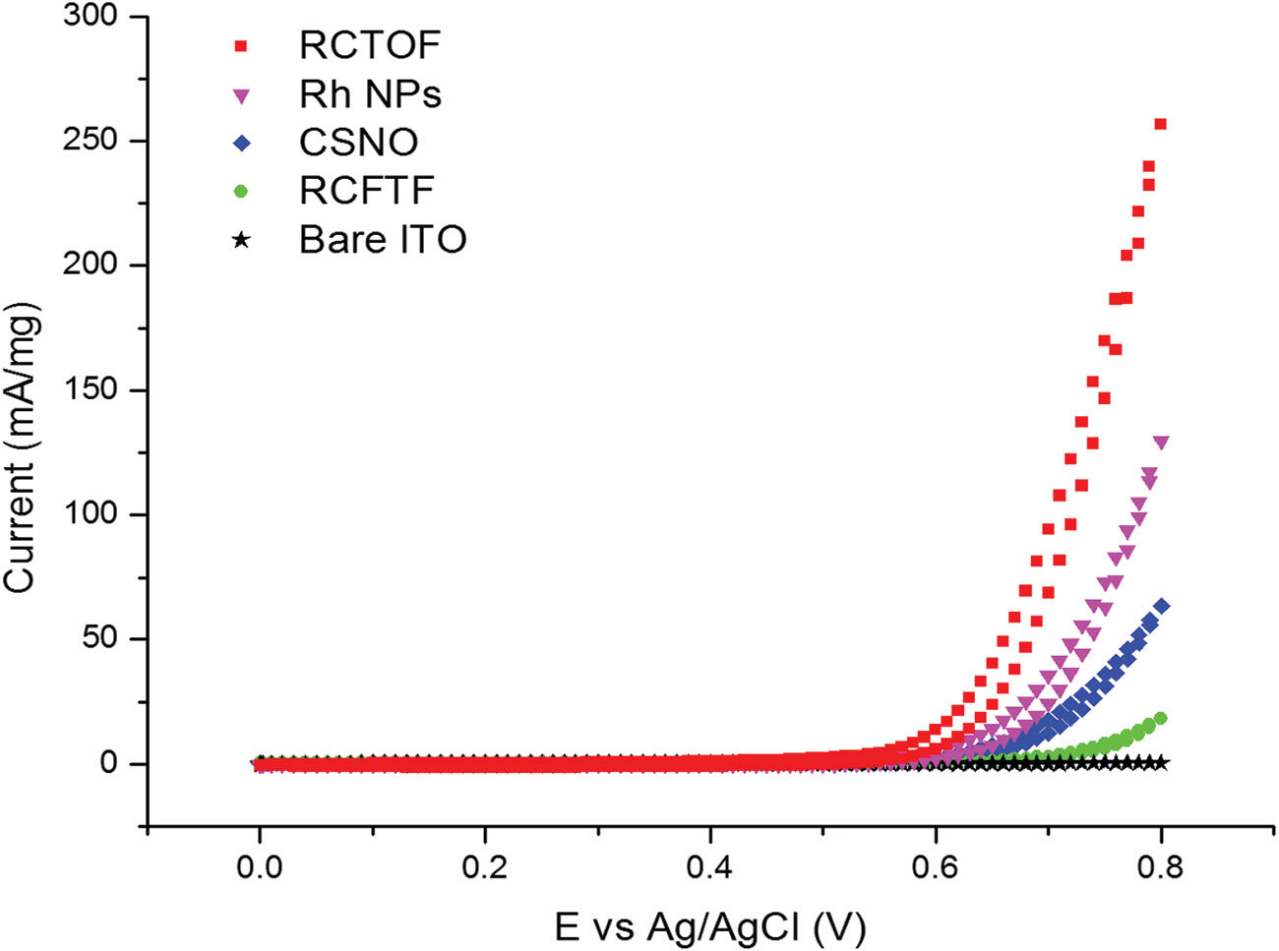
Cyclic voltammograms of RCTOF/ITO, Rh particles/ITO, CSNO/ITO, RCFTF/ITO, and bare ITO electrode at the scan rate of 10 mV s^−1^ in 0.1 m NaOH.

In summary, we demonstrated one pot synthesis of a RhCu alloy truncated octahedral nanoframe, a Cu@Rh core–shell nanoparticle, and a bundle of five RhCu nanowires by understanding the crystal growth mechanism of RhCu alloy nanoparticles and by utilizing the in situ destabilization of Cu phase. The RhCu truncated octahedral nanoframe structure, in particular, exhibits a superior electrocatalytic performance toward OER to other nanostructures under alkaline condition, and the unique accessibility and high surface area per volume of the 3D nanoframe structure seem to be responsible for its excellent catalytic activity. A further study to extend the synthetic approach to other nanoframe structures and to fine‐tune their catalytic performance toward OER under alkaline condition is currently under way.

## Experimental Section

*Preparation of RCTOF*: A slurry of Rh(acac)_3_ (0.02 mmol), Cu(acac)_2_ (0.02 mmol), stearic acid (0.04 mmol), and oleylamine (15 mmol) was prepared in a 100 mL Schlenk tube with magnetic stirring. After placing the solution under vacuum at 25 °C for 10 min, the solution was charged with 1 atm CO. Then the Schlenk tube was directly placed in a hot oil bath, which was preheated to 250 °C. After heating at the same temperature for 30 min, the reaction mixture was cooled down to room temperature with magnetic stirring. The reaction mixture, after being cooled down to room temperature and being added 15 mL toluene and 20 mL ethanol, was centrifuged at 3500 rpm for 5 min. The resulting precipitates were further purified two times by washing with 20 mL ethanol and 10 mL toluene.

*Preparation of Cu@Rh Core–Shell Nanoctahedron (CSNO)*: A slurry of Rh(acac)_3_ (0.08 mmol), Cu(acac)_2_ (0.02 mmol), stearic acid (0.04 mmol), and oleylamine (15 mmol) was prepared in a 100 mL Schlenk tube with magnetic stirring. After placing the solution under vacuum at 25 °C for 10 min, the solution was charged with 1 atm CO. Then the Schlenk tube was directly placed in a hot oil bath, which was preheated to 250 °C. After heating at the same temperature for 30 min, the reaction mixture was cooled down to room temperature with magnetic stirring. The reaction mixture, after being cooled down to room temperature and being added 15 mL toluene and 20 mL ethanol, was centrifuged at 3500 rpm for 5 min. The resulting precipitates were further purified two times by washing with 20 mL ethanol and 10 mL toluene.

*Preparation of RhCu Hollow Nanocube*: A slurry of Rh(acac)_3_ (0.02 mmol), Cu(acac)_2_ (0.02 mmol), stearic acid (0.04 mmol), 1,2‐hexadecanediol (0.02 mmol), and oleylamine (15 mmol) was prepared in a 100 mL Schlenk tube with magnetic stirring. After placing the solution under vacuum at 25 °C for 10 min, the solution was charged with 1 atm CO. Then the Schlenk tube was directly placed in a hot oil bath, which was preheated to 250 °C. After heating at the same temperature for 30 min, the reaction mixture was cooled down to room temperature with magnetic stirring. The reaction mixture, after being cooled down to room temperature and being added 15 mL toluene and 20 mL ethanol, was centrifuged at 3500 rpm for 5 min. The resulting precipitates were further purified two times by washing with 20 mL ethanol and 10 mL toluene.

*Preparation of a Bundle of Five Nanowires with Both Ends Tied (RCFTF)*: A slurry of Rh(acac)_3_ (0.02 mmol), Cu(acac)_2_ (0.02 mmol), stearic acid (0.04 mmol), 1,2‐hexadecanediol (0.02 mmol), and oleylamine (15 mmol) was prepared in a 100 mL Schlenk tube with magnetic stirring. The tube, placed in the oil bath, was heated to 100 °C and purged with Ar gas for 4 h. Then the Schlenk tube was directly placed in a hot oil bath, which was preheated to 250 °C. After heating at the same temperature for 30 min, the reaction mixture was cooled down to room temperature with magnetic stirring. The reaction mixture, after being cooled down to room temperature and being added 15 mL toluene and 20 mL ethanol, was centrifuged at 3500 rpm for 5 min. The resulting precipitates were further purified two times by washing with 20 mL ethanol and 10 mL toluene.

*Electrochemical Measurement*: Before measuring electrocatalytic activity of nanoparticles, nanoparticles were treated with acetic acid to remove organic ligands. The synthesized nanoparticles were put into vial with 2 mL Ethanol and 2 mL acetic acid and stirred for 3 h at 60 °C in oil bath. After acetic acid treatment, the nanoparticles were separated and washed by centrifuge and dried in desiccator for 24 h.

ITO electrodes were obtained from Corning (Daegu, Korea) and pretreated by dipping in 1 m HCl for 10 min. To prepare nanoparticle‐modified electrodes, 70 μL of an ethanol solution containing 100 μg mL^−1^ each nanoparticle was dropped onto ITO electrodes (1 cm × 2 cm). Afterward, the electrodes were dried at 80 °C for 10 min. Electrochemical measurements were carried out using CHI 617 (CH Instruments, Inc., Austin, TX, USA). A Teflon electrochemical cell was assembled with an ITO working electrode, an Ag/AgCl (3 m NaCl) reference electrode, and a Pt counter electrode. The exposed electrode area was 0.28 cm^2^. Every cyclic voltammogram corresponds to the second scan of two successive cyclic scans.

## Supporting information

As a service to our authors and readers, this journal provides supporting information supplied by the authors. Such materials are peer reviewed and may be re‐organized for online delivery, but are not copy‐edited or typeset. Technical support issues arising from supporting information (other than missing files) should be addressed to the authors.

Supplementary
