# Supplementary material for: RhCu 3D Nanoframe as a Highly Active Electrocatalyst for Oxygen Evolution Reaction under Alkaline Condition
Source: Adv Sci (Weinh). 2015 Sep 25;3(4):1500252. doi: 10.1002/advs.201500252 (PMC5054855; doi:10.1002/advs.201500252)
Supplement: Supplementary file 1 — Supplementary [file ADVS-3-0f-s001.pdf]

Copyright WILEY-VCH Verlag GmbH & Co. KGaA, 69469 Weinheim, Germany, 2015.

ADVANCED SCIENCE
Open Access

Supporting Information

for *Adv. Sci.*, DOI: 10.1002/advs. 201500252

RhCu 3D Nanoframe as a Highly Active Electrocatalyst for Oxygen Evolution Reaction under Alkaline Condition

Jongsik Park, Jongchan Kim, Yoojin Yang, Donghwan Yoon, Hionsuck Baik, Seungjoo Haam, Haesik Yang, and Kwangyeol Lee**

Supporting Information

Title RhCu 3-D nanoframe as a highly active electrocatalyst for oxygen evolution reaction under alkaline condition

Jongsik Park, Yoojin Yang, Donghwan Yoon, Jongchan Kim, Hionsuck Baik, Seungjoo Haam, Haesik Yang and Kwangyeol Lee**

1. Experimental details

Reagents

Rh(acac)$_3$ (99.9%) was purchased from STREM, Cu(acac)$_2$ (99.99+%), stearic acid (95%), 1,2-hexadecanediol (technical grade, 90%), and oleylamine (technical grade, 70%) was purchased from Sigma-Aldrich. All reagents were used as received without further purification.

Material Characterizations

Transmission electron microscopy (TEM) and high-resolution TEM were performed on a TECNAI G2 20 S-Twin operated at 200kV and TECNAI G2 F30 operated at 300 kV. Elemental mapping and energy dispersive X-ray spectra (EDX) were obtained with a FEI Titan Cubed 60-300 with Chemi-STEM technology and a JEOL ARM200F Cs STEM. X-ray diffraction (XRD) patterns were collected with a Rigaku Ultima III diffractometer system using a graphite-monochromatized Cu-Kα radiation at 40 kV and 30 mV.

2. Analysis of experimental data

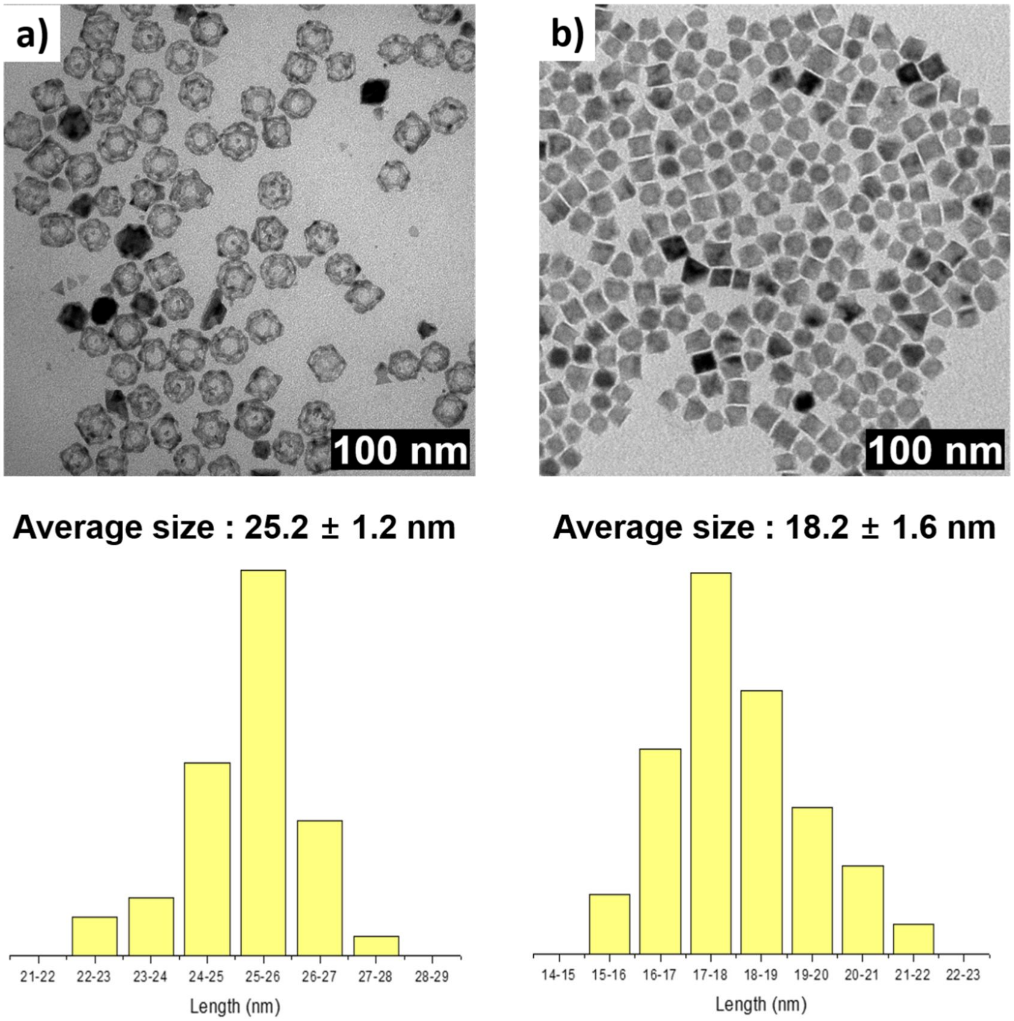

Figure S1. Size distribution data of RCTOF and CSNO. TEM images of a) RhCu truncated octahedron nanoframes and b) Cu@Rh octahedrons. Histograms show the size distributions of the RhCu truncated octahedral nanoframe and Cu@Rh nanooctahedron with the average sizes of 25.2 nm and 18.2 nm, respectively.

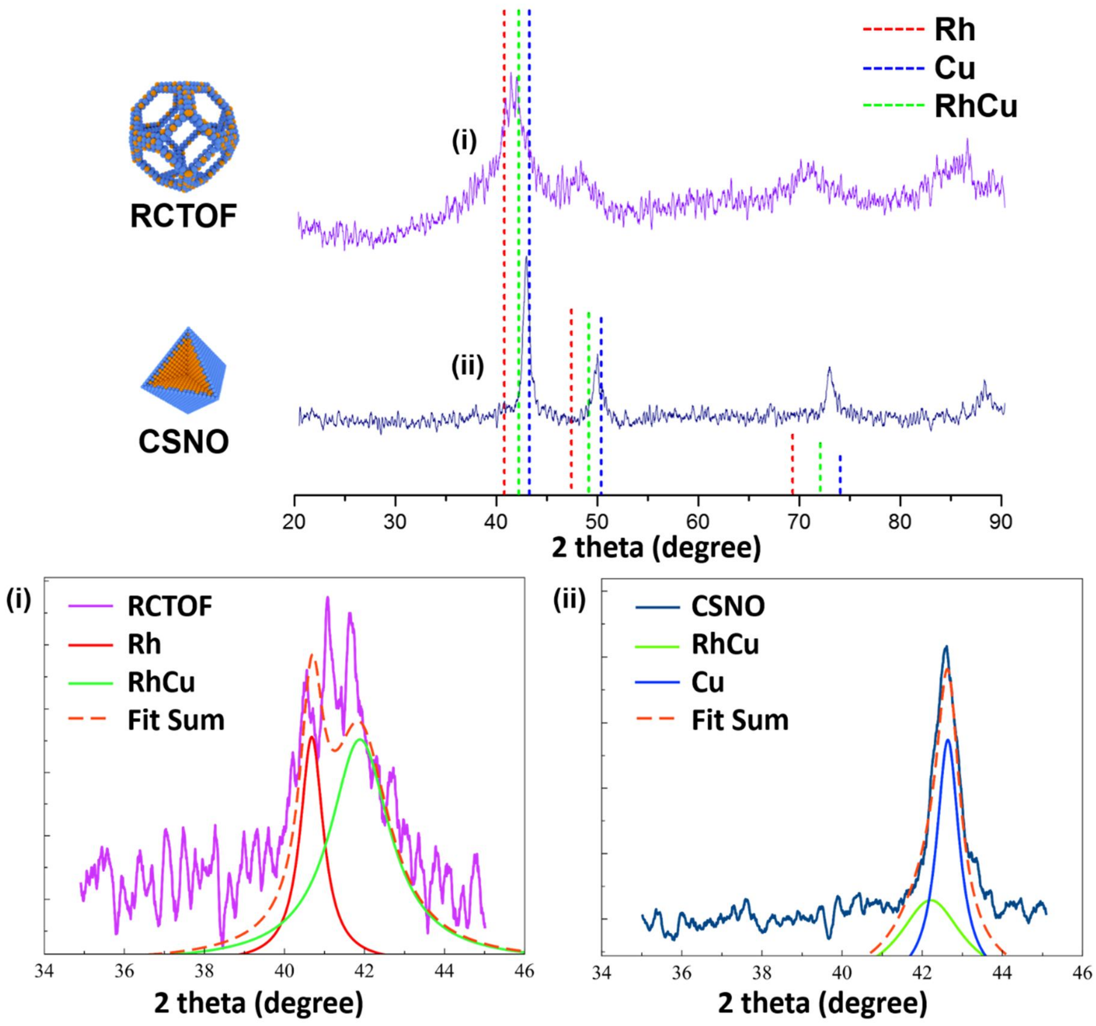

Figure S2. X-ray diffraction patterns of RCTOF and CSNO compared with Rh peaks (JCPDS card no.01-088-2334), Cu peaks (JCPDS card no.01-089-2838), and RhCu peaks (JCPDS card no.03-065-9051). The XRD peaks are deconvoluted in order to examine the relative contributions of Rh, Cu, and RhCu in (i) and (ii).

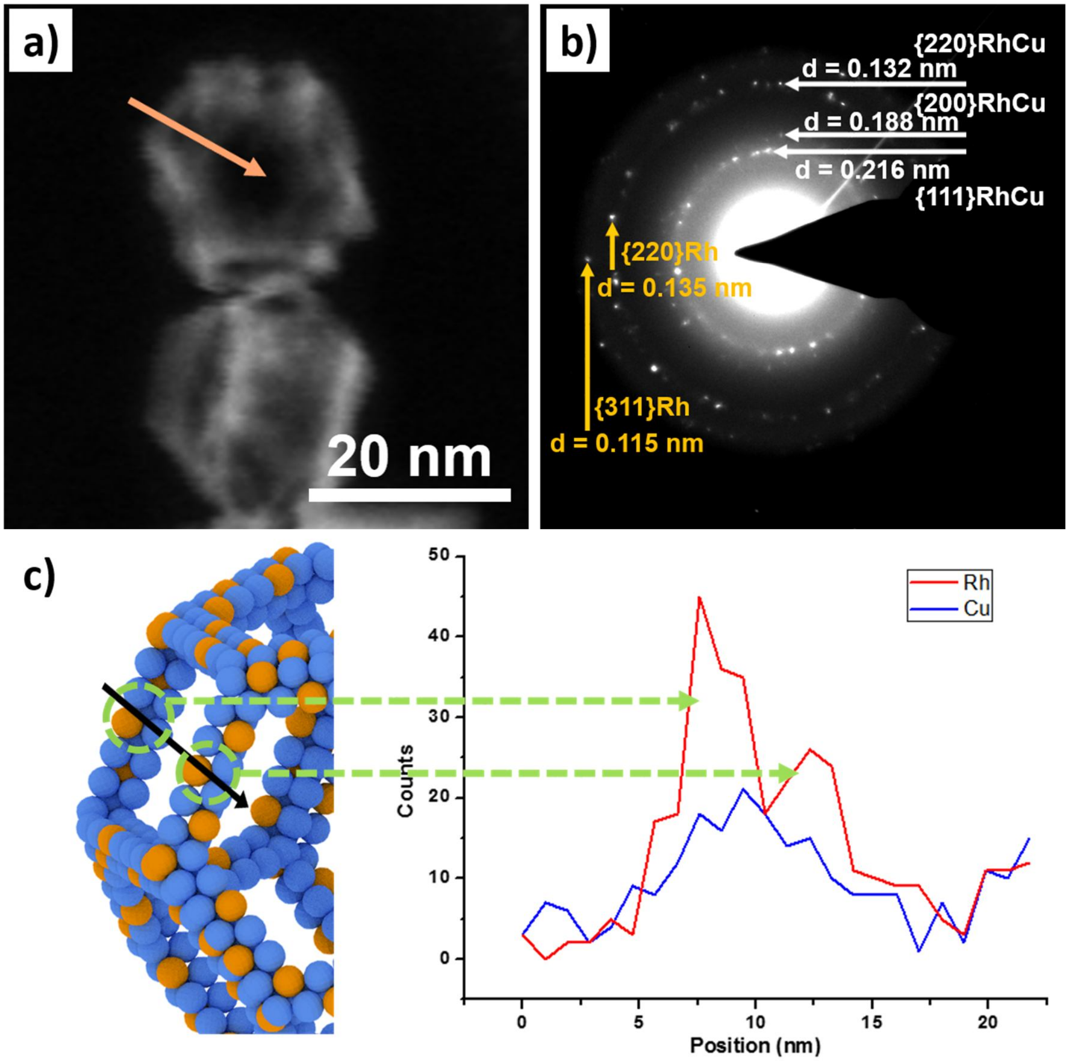

Figure S3. a) STEM image, b) ED pattern, and c) line profile data of RCTOF.

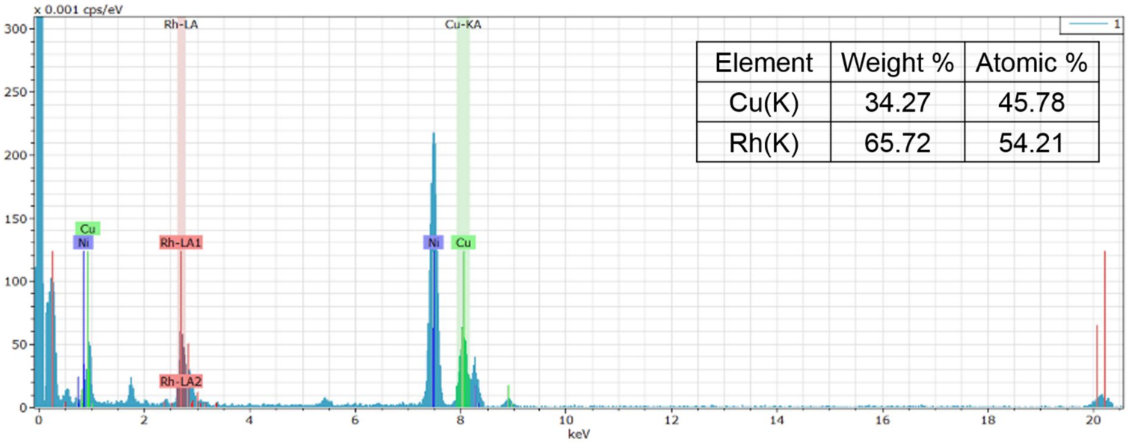

Figure S4. The energy dispersive X-ray spectrum of RCTOF.

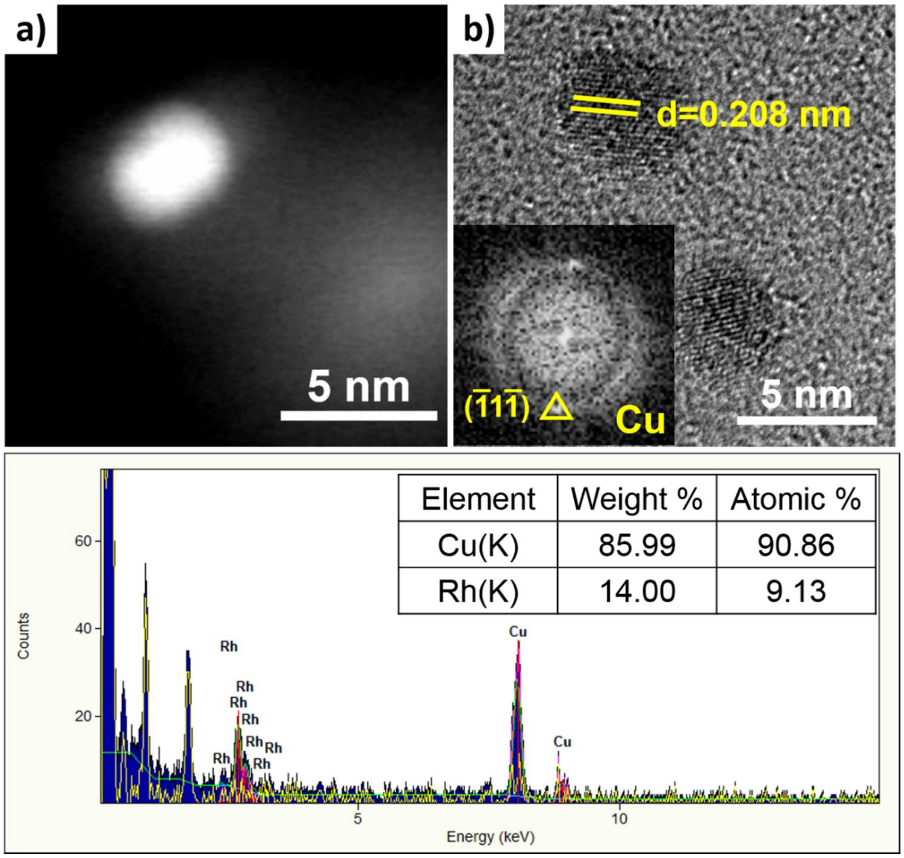

Element	Weight %	Atomic %
Cu(K)	85.99	90.86
Rh(K)	14.00	9.13

Figure S5. STEM image and HRTEM image of Cu nanoparticles collected at the reaction time of 3 min. The nanoparticle consists of 90.86% Cu and 9.13% Rh as determined by energy dispersive X-ray study (EDX).

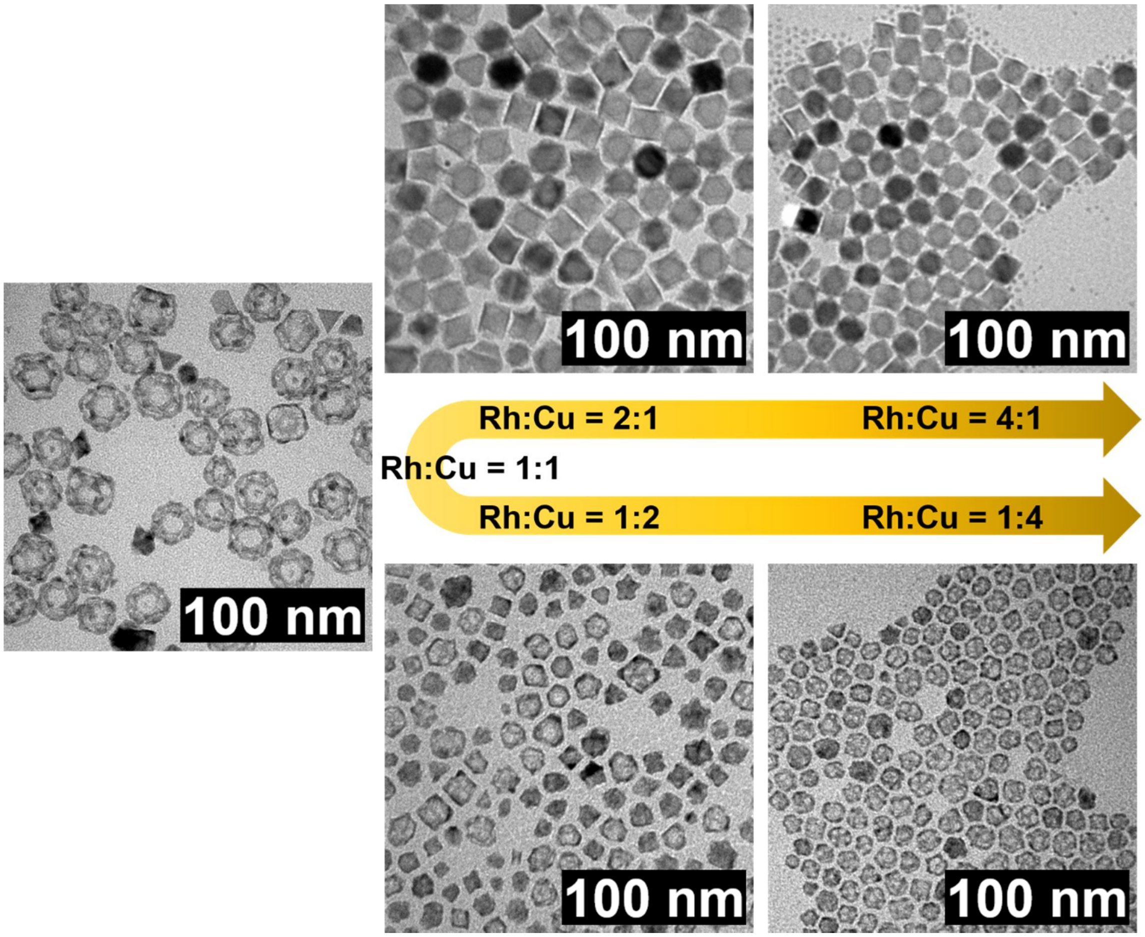

Figure S6. TEM images of Rh/Cu ratio dependent structural evolution from RCTOF to CSNO and RhCu hollow cage. The optimal ratio condition for RCTOF is 1:1.

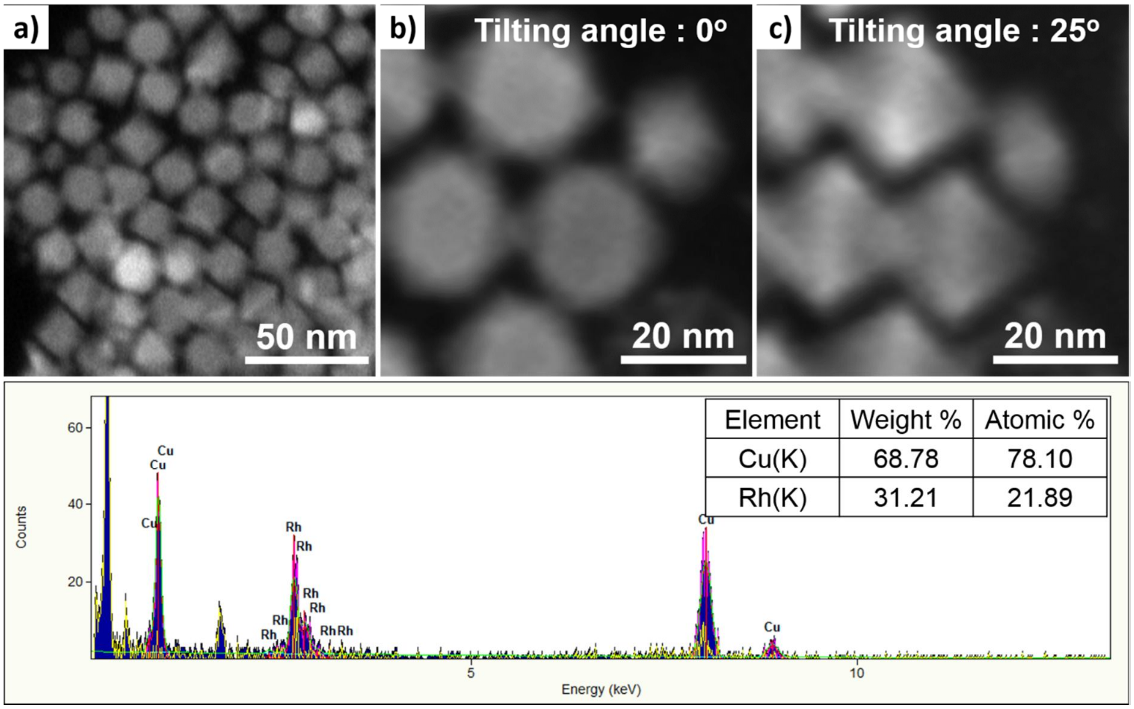

Figure S7. STEM images at different tilting angles and EDX data of CSNO. a-c) STEM images of Cu@Rh nanooctahedron structures recorded at different tilting angles. d) The energy dispersive X-ray spectrum of Cu@Rh nanooctahedron nanostructures.

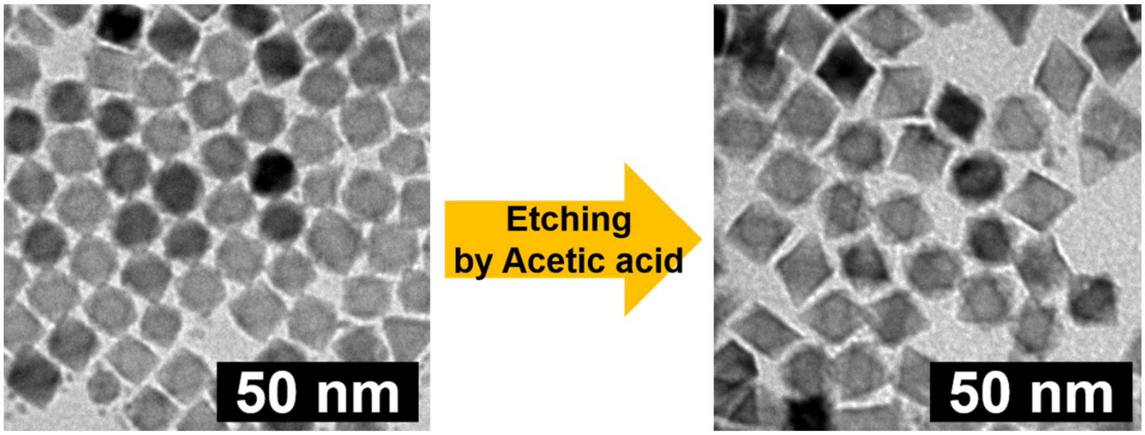

Figure S8. **TEM images of CSNO** a) before treatment acetic acid and b) after etching by acetic acid. It could be seen that the inner Cu core phase still remains even after etching.

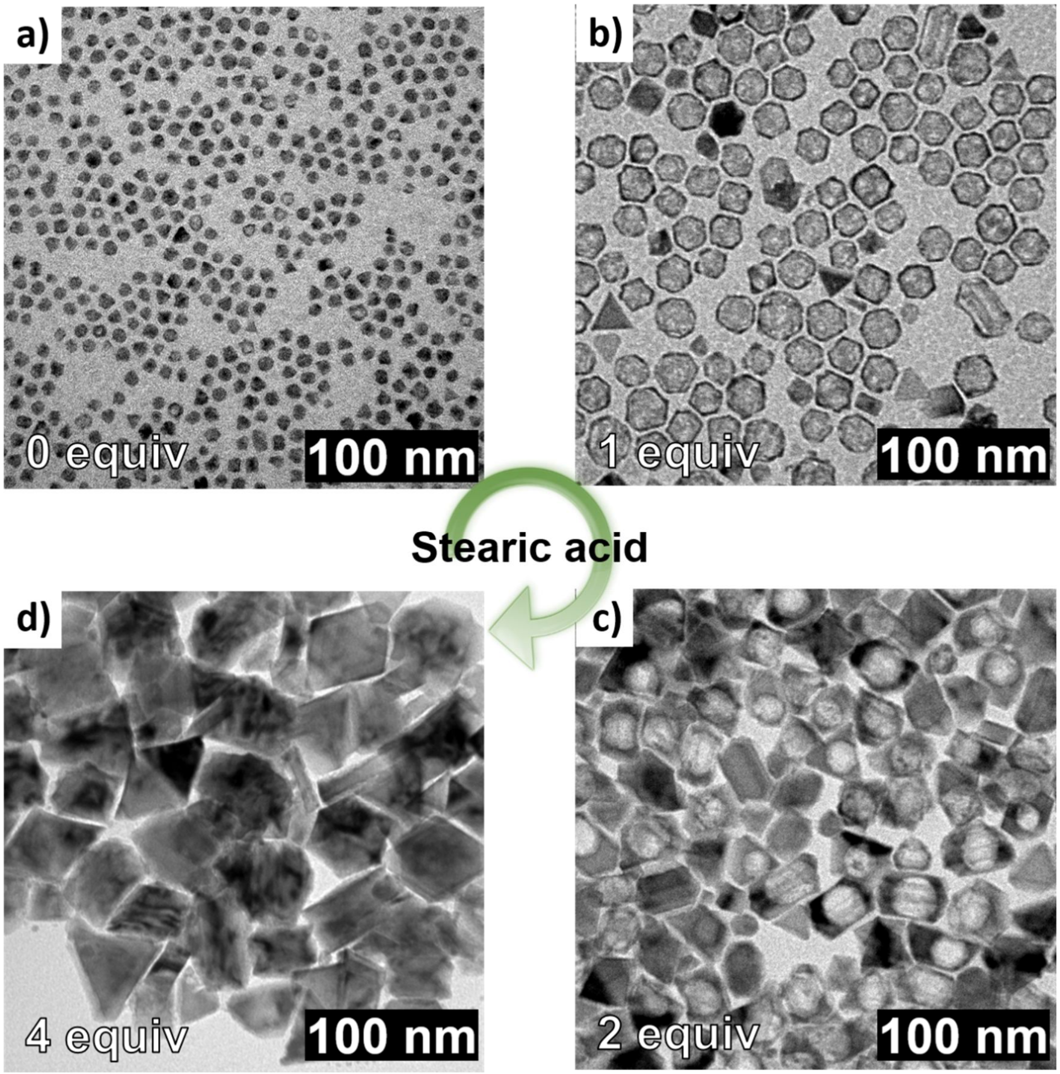

Figure S9. Structural evolution of RCTOF, dependent on the amount of stearic acid (SA). TEM images of a) small Cu nanoparticles, b) RhCu alloy hollow cage, c) RhCu alloy structure with a big hole, d) RhCu alloy nanoparticles with uncontrolled shapes.

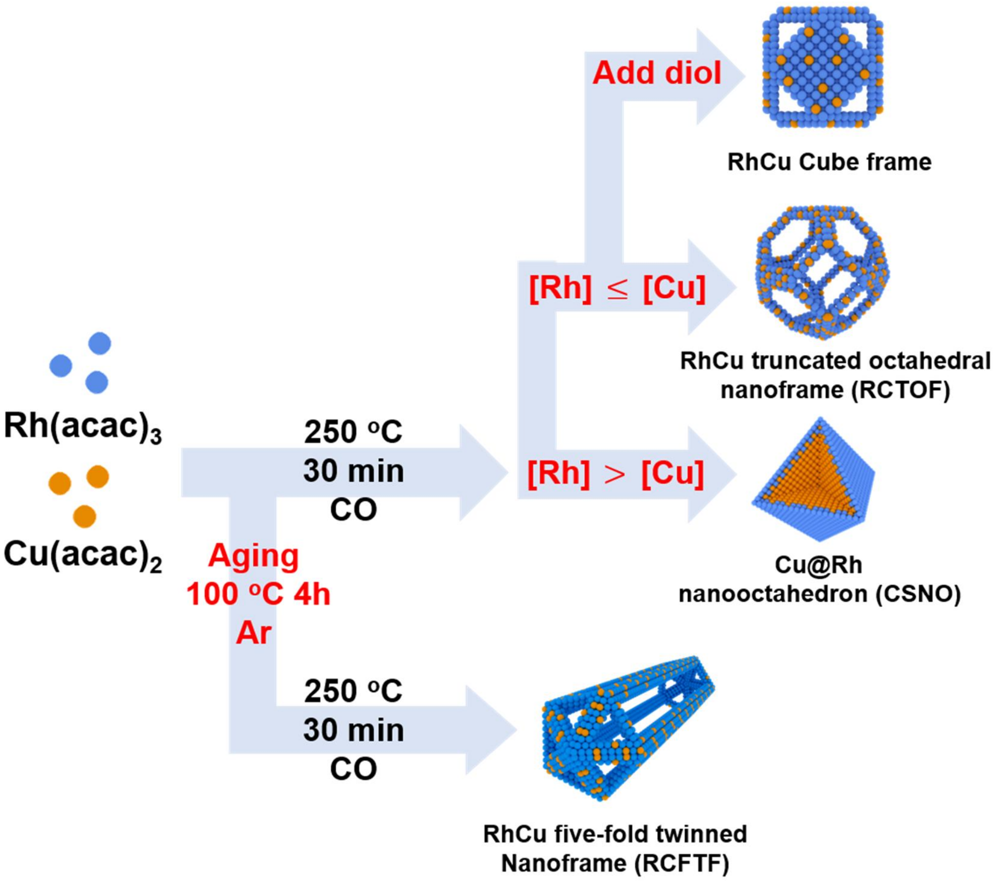

Figure S10. Overview of experimental conditions to various RhCu bimetallic nanostructures.

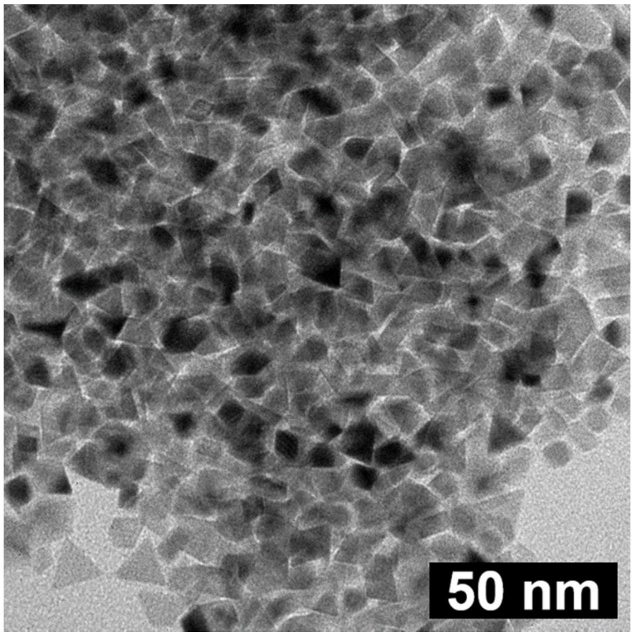

Figure S11. TEM image of Rh nanoparticles synthesized without Cu seeds. Polyhedral nanoparticles with size ranging from 10 nm to 30 nm are synthesized.

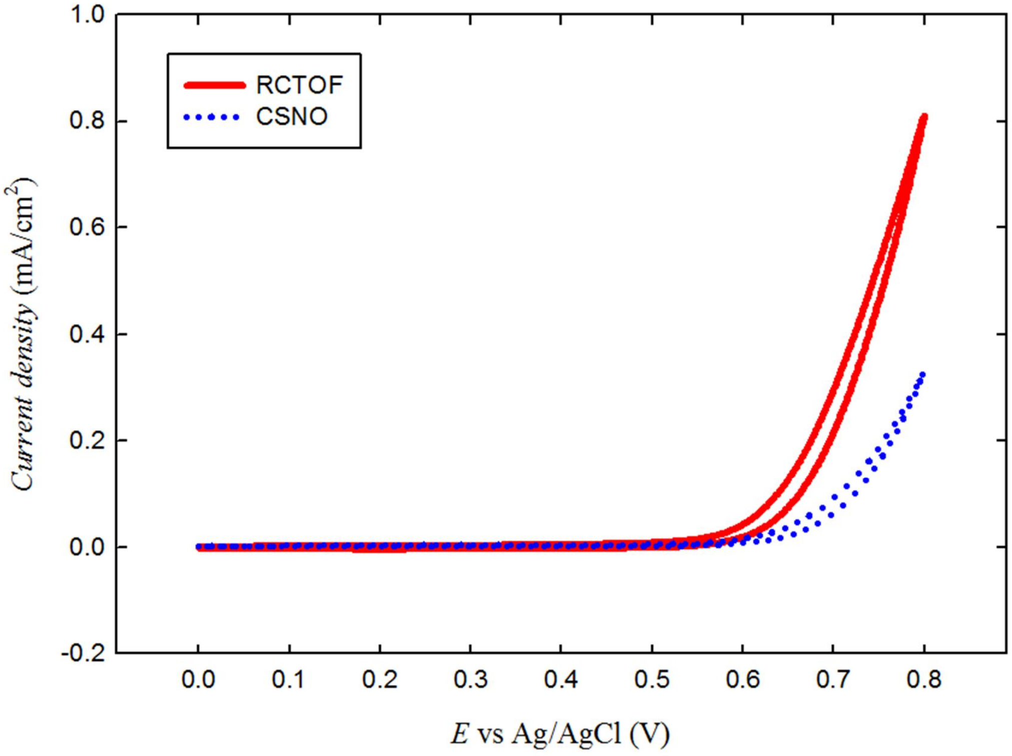

Figure S12. The specific activities of RCTOF and CSNO. It is clearly shown that the electrocatalytic activity of RCTOF is much superior to CSNO. The electrochemically active surface area (ECSA) was obtained by the equation.[1]

$$\mathrm{ECSA} = C_{\mathrm{DL}}/C_{\mathrm{s}}$$

where C_{DL} is the double-layer capacitance of a sample and C_s is the capacitance of an atomically smooth planar surface of the material per unit area in 1 M NaOH (0.040 mF cm^{-2}). C_s is given by $(i_a + i_c)/2 = \nu C_{DL}$ where i_a is the charging current obtained during the anodic scan, i_c is the charging current obtained during the cathodic scan, and ν is the scan rate. A plot of $(i_a + i_c)/2$ as a function of ν gives a straight line with a slope equal to C_{DL}.

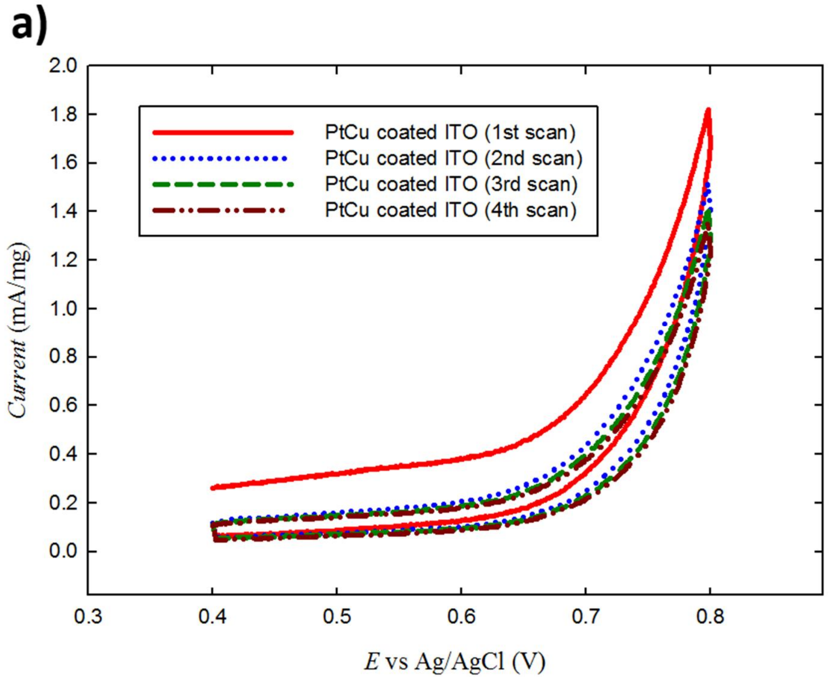

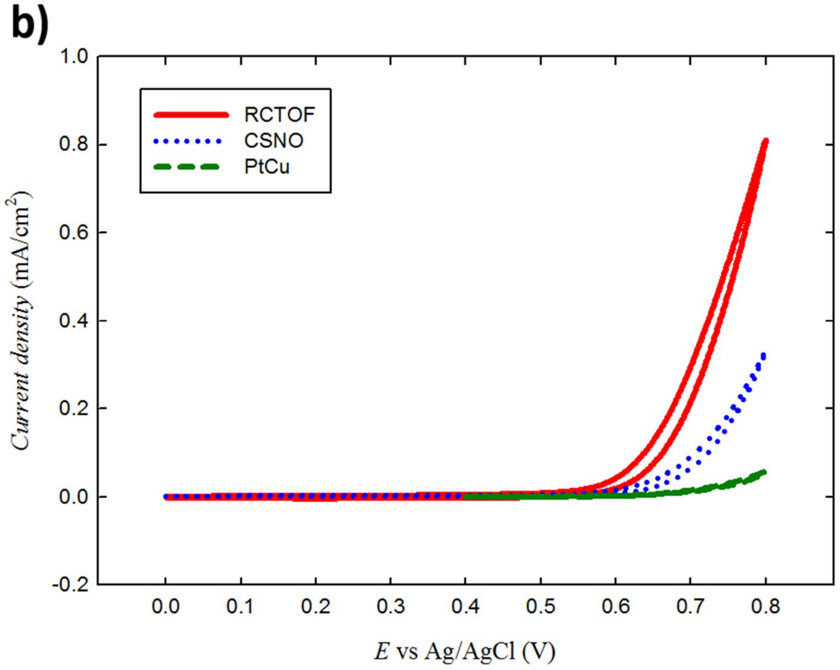

Figure S13. a) Cyclic voltammograms of PtCu hollow nanostructure at the scan rate of 10 mV/s in 0.1 M NaOH. b) The specific activities of RCTOF, CSNO, and PtCu bimetallic nanostructures.

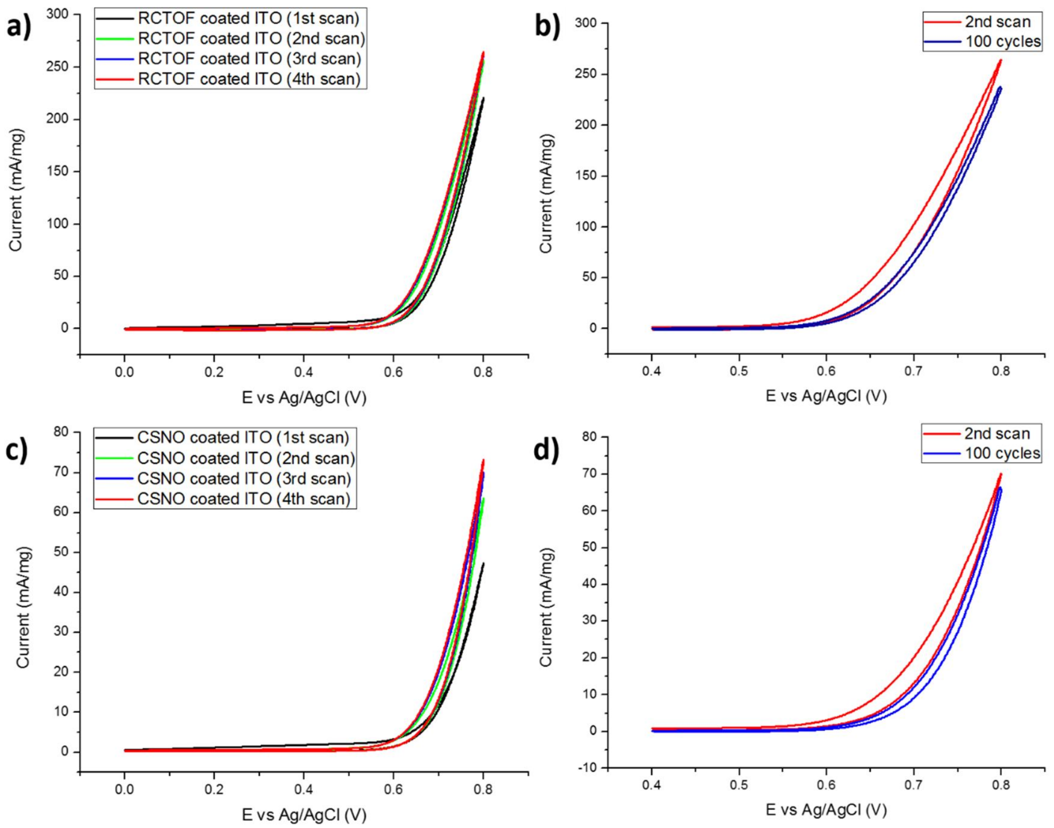

Figure S14. The current behaviour change of **RCTOF/ITO after repeated cycles** between 0.4 V and 0.8 V **a)** from 1st scan to 4th scan **and b)** from 2nd scan to 100th scan. The current behaviour change of CSNO/ITO after repeated cycles between 0.4 V and 0.8 V **c)** from 1st scan to 4th scan **and d)** from 2nd scan to 100th scan.

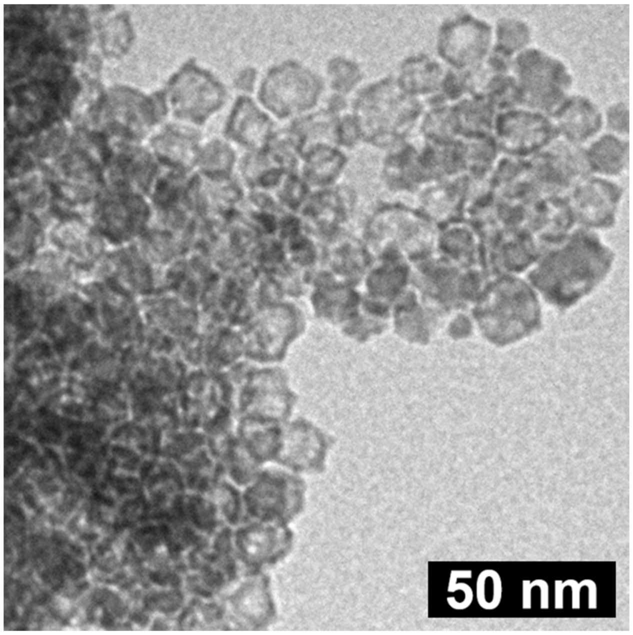

Figure S15. TEM image of RCTOF after the electrochemical measurements.

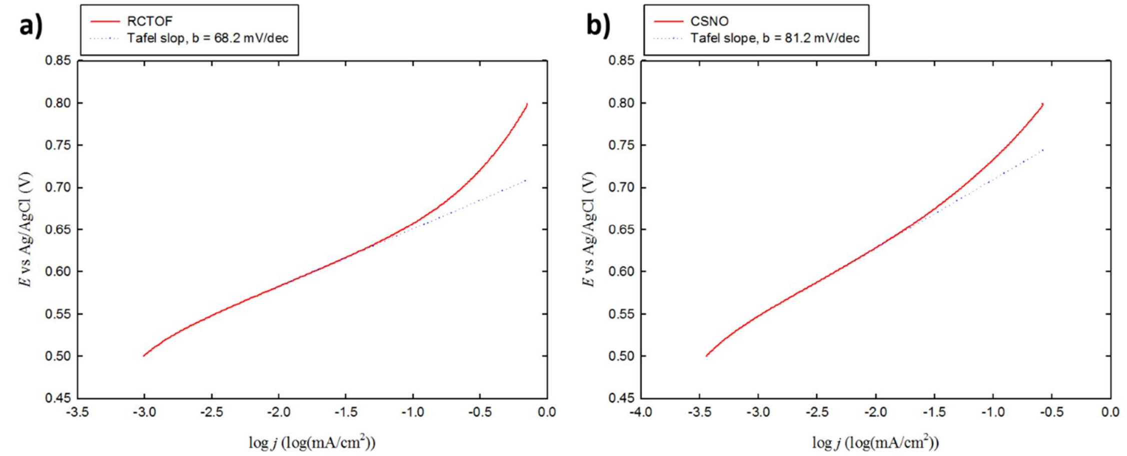

Figure S16. The Tafel curves of RCTOF and CSNO in 0.1 M NaOH. The polarization curves were obtained in 0.1 M NaOH at a scan rate of 1 mV s^{-1} with *iR* compensation.

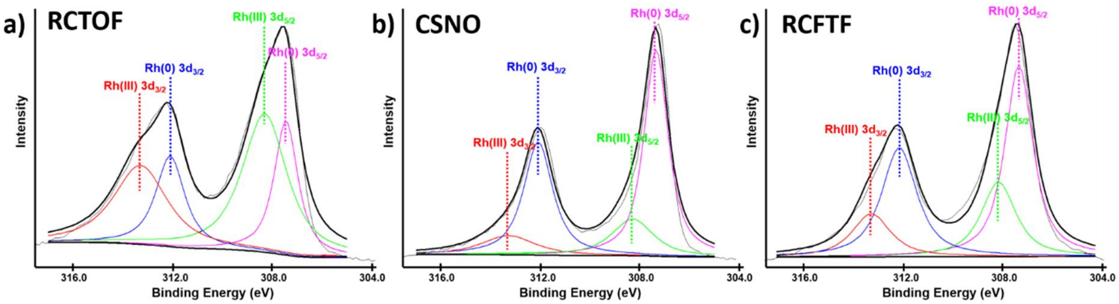

Element	Transition	Peak position (eV)	Peak width (FWHM, eV)			Peak Area (%)		
			RCTOF	CSNO	RCFTF	RCTOF	CSNO	RCFTF
Rh(0)	$3d_{5/2}$	307.4	1.2	1.2	1.3	34.2	76.1	67.8
Rh(III)	$3d_{5/2}$	308.3	2.2	2.0	1.6	65.8	23.9	32.2
Rh(0)	$3d_{3/2}$	312.1	1.4	1.4	1.6	38.5	76.4	69.8
Rh(III)	$3d_{3/2}$	313.3	2.6	2.5	1.85	61.5	23.6	30.2

Figure S17. X-ray photoelectron spectroscopy (XPS) analysis of a) Rh in RCTOF, b) Rh in CSNO, and c) Rh in RCFTF and the deconvolution results for determination of oxidation states of Rh.

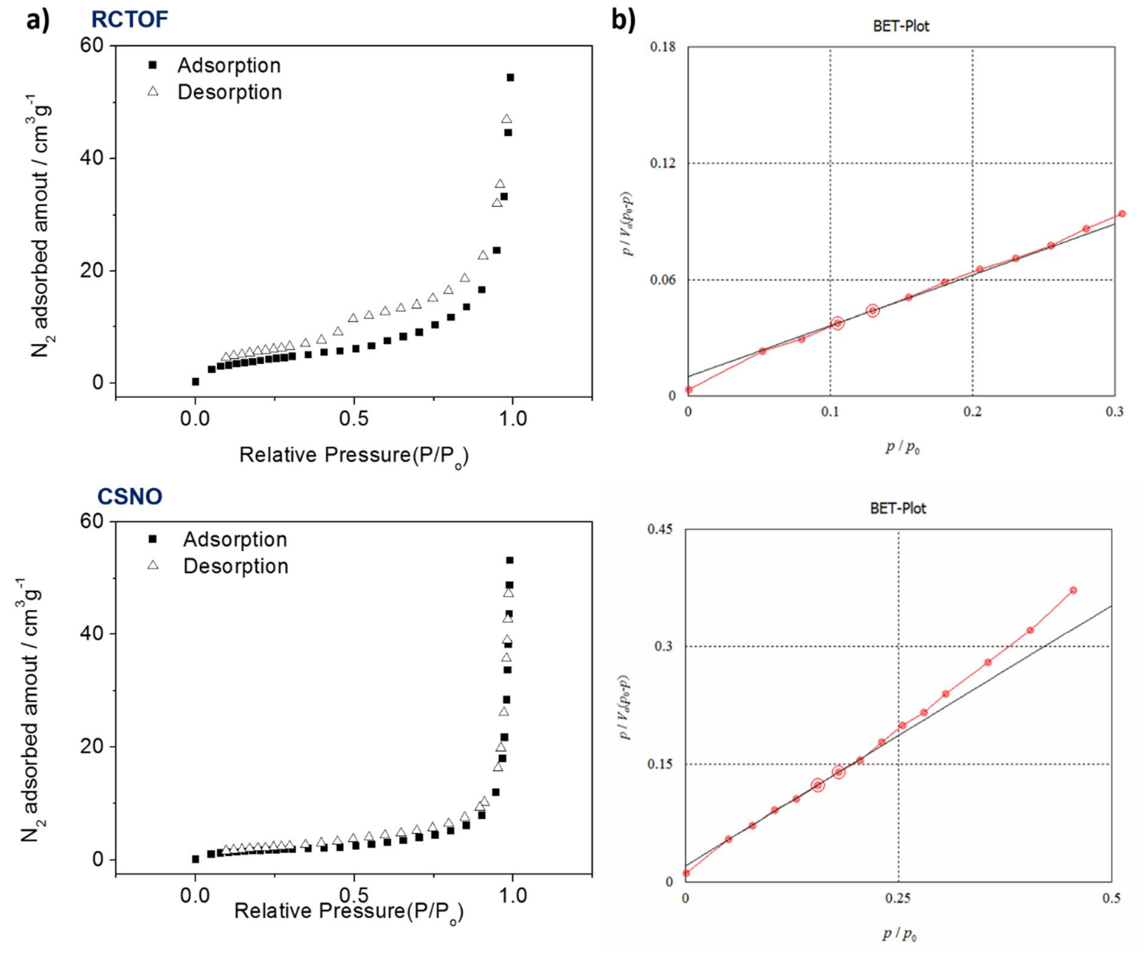

Figure S18. a) N_2 adsorption-desorption isotherms (squares, adsorption; triangles, desorption) and b) BET surface area plots of as-synthesized RCTOF and CSNO.

[1] Charles C. L. McCrory, S. Jung, J. C. Peters, T. F. Jaramillo, *J. Am. Chem. Soc.*, **2013**, 135, 16977.
